# Lignins Isolated via Catalyst-Free Organosolv Pulping from *Miscanthus x giganteus*, *M. sinensis*, *M. robustus* and *M. nagara*: A Comparative Study

**DOI:** 10.3390/molecules26040842

**Published:** 2021-02-05

**Authors:** Michel Bergs, Yulia Monakhova, Bernd W. Diehl, Christopher Konow, Georg Völkering, Ralf Pude, Margit Schulze

**Affiliations:** 1 Department of Natural Sciences, Bonn-Rhein-Sieg University of Applied Sciences, von-Liebig-Strasse 20, D-53359 Rheinbach, Germany; michel.bergs@spectral-service.de; 2 Spectral Service AG, Emil-Hoffmann-Strasse 33, D-50996 Köln, Germany; bernd.diehl@spectral-service.de; 3 Institute of Chemistry, Saratov State University, Astrakhanskaya Street 83, 410012 Saratov, Russia; yul-monakhova@mail.ru; 4 Department of Natural Sciences, University of Applied Sciences Aachen, Chemistry and Biotechnology, Heinrich-Mußmann-Strasse 1, 52428 Jülich, Germany; 5 Department of Chemistry, MS 015, Brandeis University, 415 South Street, Waltham, MA 02453, USA; ckonow@brandeis.edu; 6 Institute of Crop Science and Resource Conservation (INRES), Faculty of Agriculture, University of Bonn, Klein-Altendorf 2, D-53359 Rheinbach, Germany; g.voelkering@uni-bonn.de (G.V.); r.pude@uni-bonn.de (R.P.); 7 Field Lab Campus Klein-Altendorf, Faculty of Agriculture, University of Bonn, Campus Klein-Altendorf 1, D-53359 Rheinbach, Germany

**Keywords:** *Miscanthus x giganteus*, *Miscanthus sinensis*, *Miscanthus robustus*, *Miscanthus nagara*, lignin, monolignol ratio, low-input crops

## Abstract

As a low-input crop, *Miscanthus* offers numerous advantages that, in addition to agricultural applications, permits its exploitation for energy, fuel, and material production. Depending on the *Miscanthus* genotype, season, and harvest time as well as plant component (leaf versus stem), correlations between structure and properties of the corresponding isolated lignins differ. Here, a comparative study is presented between lignins isolated from *M. x giganteus*, *M. sinensis*, *M. robustus* and *M. nagara* using a catalyst-free organosolv pulping process. The lignins from different plant constituents are also compared regarding their similarities and differences regarding monolignol ratio and important linkages. Results showed that the plant genotype has the weakest influence on monolignol content and interunit linkages. In contrast, structural differences are more significant among lignins of different harvest time and/or season. Analyses were performed using fast and simple methods such as nuclear magnetic resonance (NMR) spectroscopy. Data was assigned to four different linkages (A: β-*O*-4 linkage, B: phenylcoumaran, C: resinol, D: β-unsaturated ester). In conclusion, A content is particularly high in leaf-derived lignins at just under 70% and significantly lower in stem and mixture lignins at around 60% and almost 65%. The second most common linkage pattern is D in all isolated lignins, the proportion of which is also strongly dependent on the crop portion. Both stem and mixture lignins, have a relatively high share of approximately 20% or more (maximum is *M. sinensis* Sin2 with over 30%). In the leaf-derived lignins, the proportions are significantly lower on average. Stem samples should be chosen if the highest possible lignin content is desired, specifically from the *M. x giganteus* genotype, which revealed lignin contents up to 27%. Due to the better frost resistance and higher stem stability, *M. nagara* offers some advantages compared to *M. x giganteus. Miscanthus* crops are shown to be very attractive lignocellulose feedstock (LCF) for second generation biorefineries and lignin generation in Europe.

## 1. Introduction

Very recently, the first thirty tons of miscanthus grass were enzymatically processed into lignocellulosic sugar and ethanol in a pre-commercial lignocellulose biorefinery [1,2,3]. The test run was part of the EU-funded project “GRACE” (GRowing Advanced industrial Crops on marginal lands for biorEfineries), a milestone for the development of second-generation biorefineries [4,5]. The results of the GRACE project emphasize the industrial suitability of the underlying technology for lignocellulose sugar production. Besides sugar production, Miscanthus-derived lignins are of potential interest as a building block for chemicals production [6,7]. Recent efforts provide an impetus for the further development of a bio-based value chain and a European bioeconomic circular economy under commercial conditions [8].

*Miscanthus* is a perennial rhizome-forming grass species from the sweet grass family (*Poaceae*). It is native to subtropical and tropical regions and comes originally from Asia. The rhizome represents the horizontally growing underground storage and wintering organ. From this, tightly clumped shoots are formed as is typical for sweet grasses [9,10]. To expand the genetic base and maximize the productivity and adaptive range of the cultures, the triploid hybrid genotype *Miscanthus x giganteus* (a cross between a diploid form of *M. sinensis* and a tetraploid form of *M. sacchariflorus)* has received increased attention both in Asia and Europe [11,12]. As a result of its hybrid genotype, *M. x giganteus* shows characteristics of both types of origin. *M. x giganteus* reaches heights of up to four meters each year and can be harvested for at least 15–20 years. Combined with a high density of shoots, this leads to very high annual production yields (approximately 25 t/ha) depending on the bioclimatic situation [13,14]. It is a sterile genotype, which makes uncontrolled spreading impossible [15]. Moreover, Miscanthus crops grow even on contaminated and abandoned soils. It is also ecologically and economically advantageous that the plants do not require fertilizers annually or to be treated with pesticides [16]. The typical harvest time of *Miscanthus* starts with the senescence of the plant in later winter. This advantageously coincides with a low moisture content (15–20%) in the plant [17]. In 2017, the European Commission decided to list *Miscanthus* as a potential crop for greening measures that will have an impact on the cultivation of this crop in many European countries [18,19]. 

In contrast to most plants, which use the C3 photosynthetic pathway, *Miscanthus* shows the C4 photosynthetic pathway. C4 plants have a high rate of CO_2_ fixation, which enables increased photosynthesis [20]. Although C4 plants only make up approx. 5% of the world’s biomass, they provide approx. 23% of the fixation of CO_2_ [21]. C4 plants generate oxaloacetate (in contrast to D-3 phosphoglycerate from C3 plants) by fixing four carbon atoms. As a result, C4 plants grow very quickly. They have a very low compensation point, so they continue photosynthesis at high light intensity when only low CO_2_ concentrations are available. In addition, the rate of photorespiration in C4 plants is significantly lower than in C3 plants, since the concentration of O_2_ in relation to CO_2_ in the cells of the C4 plants that are responsible for photosynthesis (mesophyll cells) is vanishingly small. This almost completely suppresses the respiration caused by O_2_ [22].

Significant research has previously been performed to elucidate both the composition and optimal decomposition conditions of various *Miscanthus* genotypes. Van der Weijde et al. examined eight genotypes of *M. sinensis* with different cell wall composition profiles [23]. A study including 25 *Miscanthus* genotypes was reported by da Costa et al., which also included the cell wall composition of *M. x giganteus*, *M. sacchariflorus*, *M. sinensis*, and various hybrids [24]. Other studies focused the crop yield: Iqbal et al. examined 15 Miscanthus genotypes (*M. sinensis*, *M. sacchrofloris*, *M. x giganteus* and hybrids) harvested at different times of the year (November and between January and April) over a period of five years. The harvested biomasses differ significantly depending on the harvest time. External effects such as weather or aging, on the other hand, have little effect on the constituent proportions [1,13]. There is also an effort to break down lignin enzymatically or with the help of fungi. For example, Baker et al. investigated the effects of wood rot on the biomass degradation of various Miscanthus genotypes [25]. Sonnenberg et al. examined shiitake mushrooms as a means of biomass degradation. They found that a significant breakdown of lignin (and hemicellulose) in *M. x giganteus* can also be achieved with these mushrooms [26]. However, these studies [23,24,25,26] only report lignin quantity with no isolated lignin structural data. 

**Figure 1:**
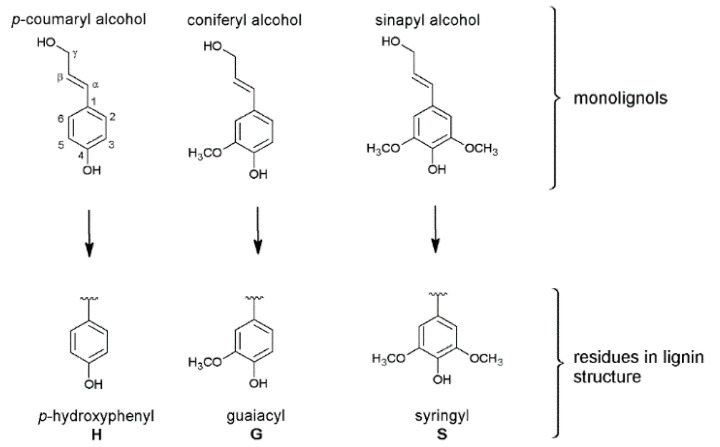
Monolignol structures: *p*-coumaryl alcohol, coniferyl alcohol and sinapyl alcohol forming the specific residues *p*-hydroxylphenyl (H), guaiacyl (G) and syringyl (S). Reprinted from [30] under open access license.

**Figure 2:**
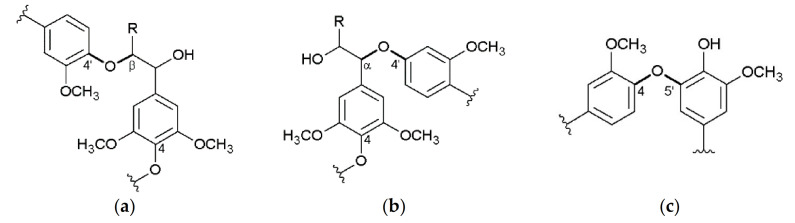
Ether linkages: β-aryl-ether (β-*O*-4′) (**a**), α-aryl-ether (α-*O*-4′) (**b**), biphenyl ether (4-*O*-5′) (**c**); R = CH_2_OH, lignin. Reprinted from [31] under open access license.

**Figure 3:**
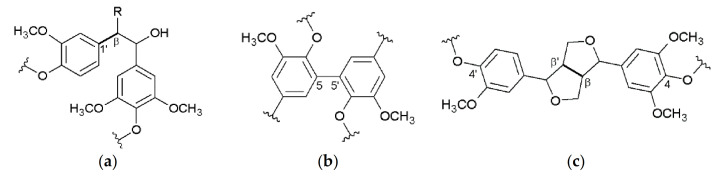
Carbon-carbon linkages: 1,2-diarylpropane (β-1′) (**a**), biphenyl (5-5′) (**b**), resinol (β-β’) (**c**); R=CH_2_OH, lignin. Reprinted from [31] under open access license.

**Figure 4:**
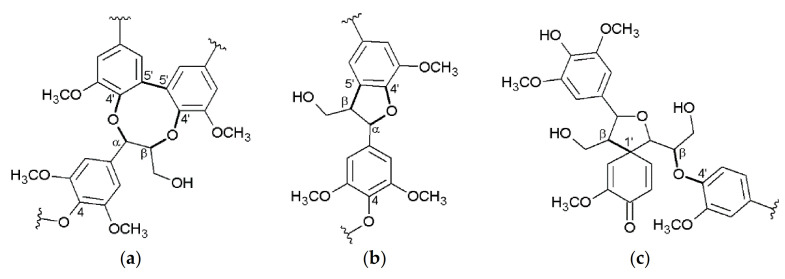
More complex linkages: dibenzodioxocin (α-*O*-4′/β-*O*-4′/5-5′) (**a**), phenylcoumaran (α-*O*-4′/β-5′) (**b**), spirodienone (β-1′/β-*O*-4′) (**c**). Reprinted from [31] under open access license.

Besides crop and cell wall composition, focus of current research is also directed toward Miscanthus-derived lignins and their detailed 3D structure, including the monolignol ratio (G, H, S, Figure 1) and corresponding interunit linkages (Figure 2, Figure 3 and Figure 4) [27,28,29,30,31]. 

Both the G/H/S ratio and the linkages strongly depend on the biomass origin (crop genotype) and biomass treatment (pulping) method for lignin isolation. Industrially, the most common pulping methods are Kraft pulping and various steam explosion techniques. However, these produce significant amounts of unusable waste [32]. On the laboratory scale, organosolv processes for *Miscanthus* pulping have become common to avoid these environmentally harmful waste products. El Hage et al. reported the structure determination and correlating effects during the pretreatment of the biomass in the ethanol organosolv digestion [33,34,35]. Chan et al. were able to isolate and partially depolymerize lignin obtained from *M. x giganteus* using a vanadium catalyzed organosolv process. Variation of the process parameter significantly influence the biomass digestion resulting in structural differences of the isolated lignins [36]. Luo et al. used a nickel-activated carbon catalyst to degrade *Miscanthus* lignin into soluble components through a methanol organosolv process under H_2_ pressure [37]. Overall, they showed that all three main components of biomass (lignin, cellulose, and hemicellulose) could be used efficiently to generate valuable chemicals. 

Vanderghem et al. compared lignins from *M. x giganteus* that they obtained from different pulping methods. The characterization was carried out by means of FTIR spectroscopy, TGA, GPC and NMR spectroscopy [38]. Lignin from *M. x giganteus* and other biomasses were studied by Timilsena et al.: the authors exposed the biomass to various pretreatment methods (e.g., autohydrolysis, treatment with 2-naphthol, enzymatic hydrolysis) and the obtained lignins have been analyzed [39]. Direct analysis of the untreated dried biomass was used by Groenewold et al. to specify the composition of *M. x giganteus*. Pyrolysis GC/MS and NMR techniques were used for structure analysis and to determine the monolignol ratio [40]. However, the native lignin structure of lignocellulose biomass is still under investigation [41,42].

The molecular weight (MW) and polydispersity (PD) are fundamental characteristics to be considered for future applications of lignin [43]. Compared to technical wood-based kraft lignin (with PD of 2.6 to 6.5 depending on biomass origin and pre-treatment conditions), the *Miscanthus*-derived lignins presented here exhibit lower PD (below 1.7) [43]. In two previously published studies [44,45], we showed the influence of crop genotype and harvesting season on MW and PD. An increase of MW was observed for stem-derived lignins of *M. x giganteus* and *M. nagara* from September to April [30,45]. Furthermore, the influence of biomass particle size on MW and PD has been investigated for different low-input crops including *M. x giganteus* [31]. Currently, diffusion ordered spectroscopy (DOSY) NMR is used and combined with size exclusion chromatography (SEC) to obtain further information on MW and PD as shown for lignin [46] and polysaccharides [47].

In this mini-review, we present a comparative study on lignins isolated from six different *Miscanthus* genotypes including *M. x giganteus* (Gig17, Gig34, Gig35), *M. sinensis* (Sin2), *M. robustus* (Rob4), and the winterhard hybrid *M. nagara* (NagG10). Leaves and stems were harvested separately from three harvests (December 2014, April 2015, and September 2015). The lignin samples were extracted from all biomasses using a catalyst-free organosolv pulping process and analyzed regarding the lignin content, monolignol composition (G, H, S) and corresponding monolignol linkages. Parts of the original data set have recently been published in previous works [30,31,44,45]. In addition, original data obtained for *M. sinensis* and *M. robustus* are presented and discussed in comparison to results found for *M. x giganteus* and *M. nagara*. Based on this information, the potential for different genotypes to serve as industrial crops for lignin isolation and utilization is discussed.

## 2. Biomass Leaf-To-Stem Ratio, Chemical Composition, Lignin and Ash Content 

### 2.1. Leave-To-Stem Ratio and Chemical Composition of the Miscanthus Biomass

**Figure 5:**
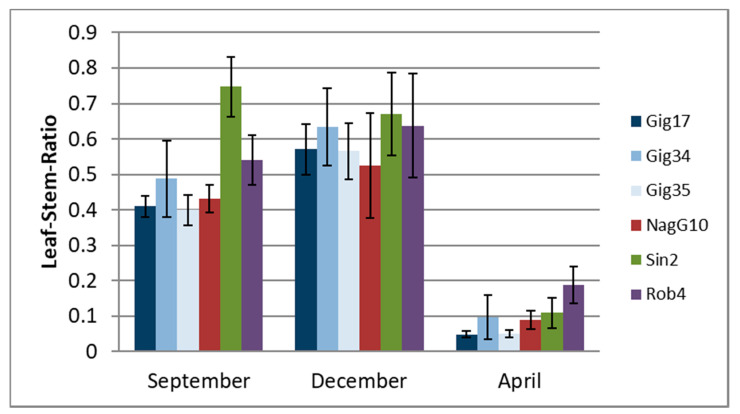
Leaf *versus* stem content (weight ratio of dry matter) of different *Miscanthus* genotypes: *M*. *x giganteus* (Gig17, Gig34, Gig35), *M*. *nagara* (NagG10), *M*. *sinensis* (Sin2), and *M*. *robustus* (Rob4) harvested in September (09/15), December (12/14), and April (04/15), respectively, arranged to follow the seasonal order from autumn to spring. Reprinted from [45] under open access license. Error bars are standard deviation of triplicates.

First, the seasonal influence on plant constitution was examined. Stem and leaf samples for lignin analysis were harvested in December 2014 and in April and September 2015 to compare across different years and harvest times. Plant constitution analysis showed significant differences regarding the biomass amount with respect to the year and harvest date (Figure 5).

The data in Figure 5 was arranged to follow the seasonal order from autumn to spring: In early autumn (September), the leaf content did not reach the maximum (except for *M*. *sinensis*) when compared to the December harvest. During winter, the plants lose their leaves, which results in an extremely low leaf versus stem ratio in the April harvest. The highest values were found for *M*. *robustus* (Rob4) [45].

In Table 1 and Table 2, chemical composition (according to NREL procedures) is Scheme 1. and stem (Table 2) samples from the April 2015 harvest. Structural carbohydrates in plants in general include glucan, xylan, galactan, arabinan and mannan. The total lignin content is derived from acid-soluble lignin (ASL) and acid-insoluble lignin (AIL). If one compares the dry matter, which is determined when the sample is heated at just over 100 °C, to the mass consistency of both the leaf and stem samples there are no significant differences between the two measures. An average dry matter content of 91.16% shows that the samples have already been deprived of a great deal of moisture by the previous treatment (storage, drying). The ash content shows the proportion of inorganic (mineral) components. This is determined gravimetrically from the residues during the targeted combustion of the biomass. Here, there are clear differences between leaf and stem samples. While the ash content in the stem is comparatively low at an average of 2.6%, the leaf samples have more than twice as much ash content at 5.7%. This indicates that more minerals are stored in the leaves than in the rest of the plant.

The results obtained are in good accordance with those determined for the biomass ash content and correspond to other literature data. Wahid et al. reported ash contents from 2.6 to 4.0% for *M. x giganteus* stem samples and 1.5 to 3.4% for *M. sinensis* stems. For leaf samples, ash contents vary between 3.9–7.2 for *M. x giganteus* and 3.3–5.0% for *M. sinensis* [48]. For the plant as a whole, ash contents of 1.9 to 2.3% were determined [49,50]. The average ash content of acid-insoluble residues (AIR) is 0.9% and shows that there are hardly any mineral impurities in it.

Comparing the results of the leaf samples (Table 1), it is noticeable that all genotypes except NagG10 have a lignin content of around 25% (mean: 24.5%). NagG10 drops slightly at about 22.5%. For the sugars, Gig35 and NagG10 are particularly high in glucose, which would be of interest for their further processing. The hemicellulose (shown here in parts xylan, galactan, arabinan and mannan) of the hybrid genotypes Gig17, Gig34, Gig35 and NagG10 consists mostly of xylan with small proportions of arabinan. For the pure genotypes Sin2 and Rob4, small amounts of galactan and mannan were detected, which makes the hemicellulose of these genotypes less homogeneous.

For the stem samples (Table 2), the following picture emerges: All genotypes show an average lignin content of 25.6% (i.e., slightly higher than for leaf samples), with Gig35 having the highest lignin content at 27.1%, followed by the hybrid genotypes Gig17, Gig34 and NagG10; the lowest lignin content shows Sin2 and Rob4. The sugar distribution in the stem samples shows a significantly higher glucan content compared to the leaves, caused by the higher content of cellulose in the stems. There is no special classification for hemicellulose: in all samples, xylan dominates with a small proportion of arabinan; however, mannan is another hemicellulose component in Sin2, Rob4 and Gig35. The traces of galactan found in Gig35 are 0.2%, below the deviation of ±0.4%.

The lignin content slightly differs depending on the crop genotype with lowest values (22.5%) for *M. nagara* up to 27% for *M. x giganteus*, which agrees with results reported by other groups [49,50,51,52]. For the most common Miscanthus genotype *M. x giganteus*, cellulose content varies usually between 40–50%. Hemicellulose is also largely composed of xylan with low shares of arabinan. In addition, mannan and galactan are only found at lower weight percent ratios (max 2.1%) [49,50].

### 2.2. Dry Matter

**Figure 6:**
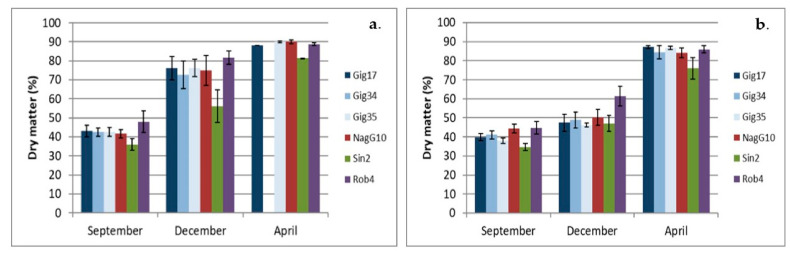
Dry matter of the leaves (**a**) and stems (**b**) of all six genotypes harvested in September (09/15), December (12/14) and April (04/15), respectively.

A comparison of the dry matter in the leaf samples (Figure 6a) shows that in total they have apparently not fully matured in the September harvest, as all samples have a dry matter of less than 50%. This changes significantly for the December harvest, as significantly drier samples are obtained here (except for Sin2). These even increase to almost 90% towards the April harvest (no values were available for genotype Gig34). Sin2 shows a significantly lower dry matter content in the context of the individual harvests than all other genotypes.

A comparison with the stem samples, the overall picture is somewhat different (Figure 6b). In the September harvest, these are also relatively rich in moisture, but show only a minimal increase in dry matter in the December harvest. In April, almost 90% of the dry matter could be assigned to the stalks for all genotypes. For all three harvests, Sin2 shows the lowest dry matter in the stalk.

### 2.3. Ash Content

**Figure 7:**
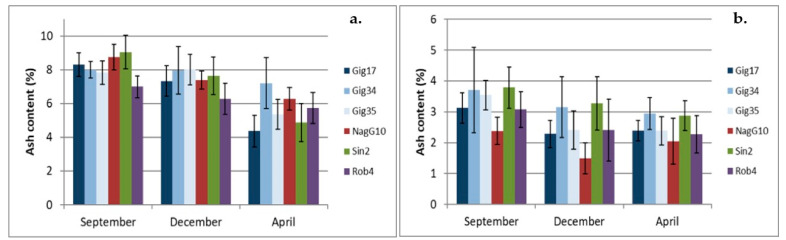
Ash content of the leaves (**a**) and stems (**b**) of the six M. genotypes harvested in September (09/15), December (12/14) and April (04/15), respectively.

For the three harvests in which the plant components were separated from each other during harvest, the ash content of the leaf and stem samples are compared below (Figure 7a,b). Similar to the NREL analysis, the leaf samples contain significantly more ash than the stem samples. When looking at the leaf samples alone, five out of six genotypes have the highest ash content in the September harvest. This decreases for all genotypes except for Gig35 (this initially seems to remain constant until December) over the December to April harvest, since parts of the mineral inorganic compounds are more heavily washed out after the senescence of the plant. In proportion, the Gig17 and Sin2 lose the most ash from September through December through April. The Gig34 shows the least variance here.

Overall, the stem samples show significantly lower ash contents, which also fluctuate less than in the leaf samples. Here, too, the highest values were found in the September harvest and the lowest in the April harvest with the exception of Gig17 and NagG10, which show a minimum in the December harvest and then rise again slightly towards April. 

Similar results are also described in the literature: Iqbal et al. reported the harvests of *Miscanthus* crops at different times and in different locations, but comparisons can still be made. Here too, the ash content for the miscanthus samples used drops towards spring [12].

## 3. Structure of the Isolated Miscanthus-Derived Lignins 

### 3.1. Monolignol Ratio Accoroding to NMR Spectroscopy

**Figure 8:**
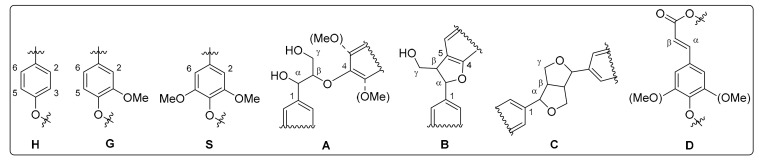
Monolignol units (**H**, **G**, **S**) and corresponding linkages (**A**: β-*O*-4 linkage, **B**: phenylcoumaran, **C**: resinol, **D**: β-unsaturated ester) of lignins according to HSQC NMR. Reprinted from [44] under open access license.

Nuclear magnetic resonance (NMR), particularly heteronuclear single quantum coherence (HSQC) NMR is used to study the detailed 3D structure of isolated lignins [53]. Spectra of the aromatic region are used to specify the lignin monomer building blocks (H, G, S), whereas the signals of the non-aromatic mainly indicate the linkage patterns (A, B, C, D) within the lignin (Figure 8) [30,31,44]. 

**Figure 9:**
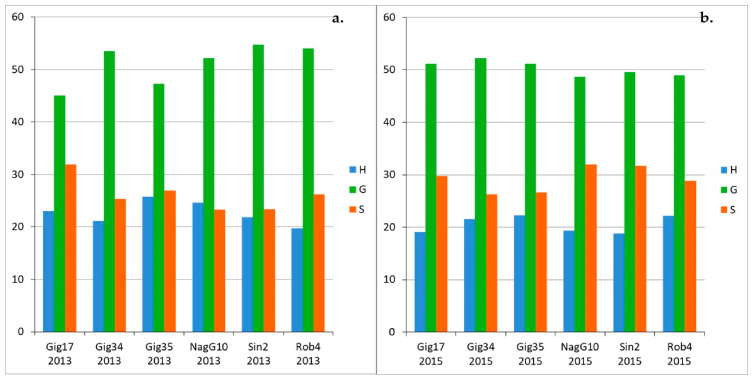
Monolignol ratios (H, G, S in %) of the crop mixtures (stem and leaves) harvested in 2013 (**a**) and 2015 (**b**) according to HSQC NMR. Precision of HSQC integration is about 5%.

**Figure 10:**
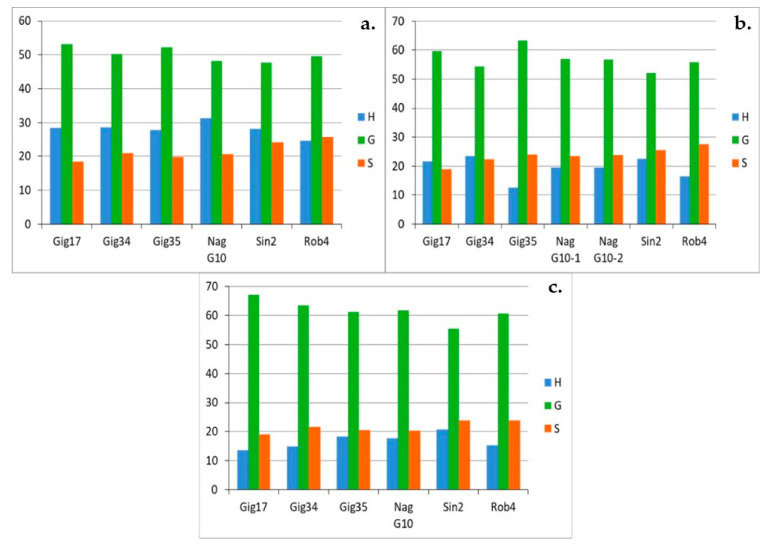
Monolignol ratios (H, G, S in %) of the leaf-derived lignins, harvested in September (09/15) (**a**), in December (12/14) (**b**) and in April (04/15) (**c**), due to HSQC NMR. NagG10-1 and NagG10-2 (Figure 10, above/right) are duplicates. Precision of HSQC integration is about 5%.

**Figure 11:**
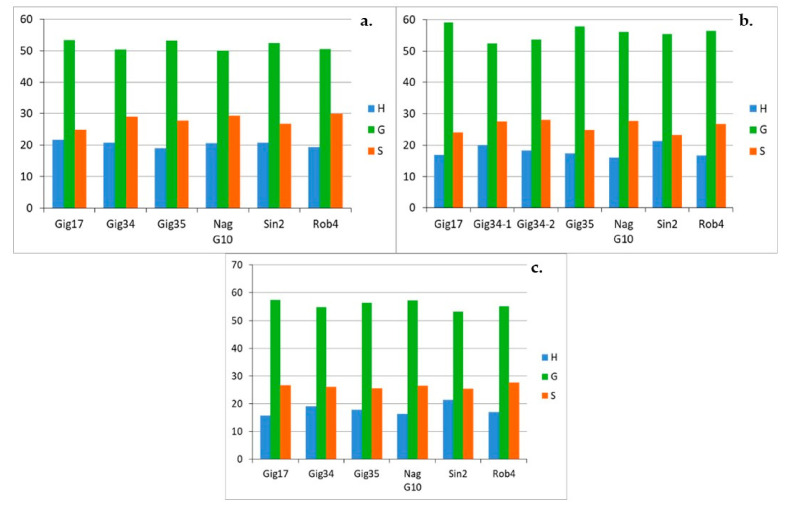
Monolignol ratio (H, G, S in %) of stem-derived lignins harvested in September (09/15) (**a**), in December (12/14) (**b**) and in April (04/15) (**c**), analyzed via HSQC NMR. Precision of HSQC integration is about 5%.

**Figure 12:**
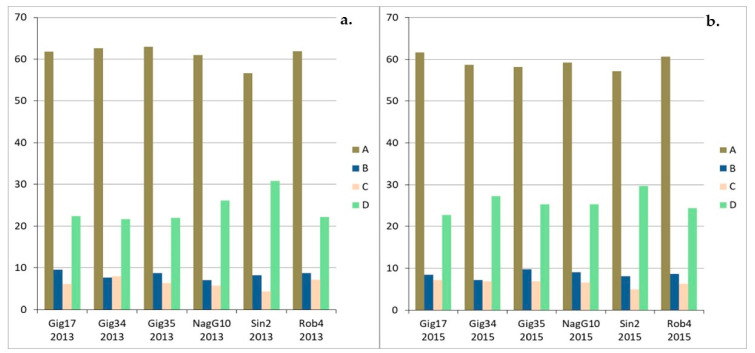
Monolignol linkages (A, B, C, D in %) of the stem/leaf mixture harvested in 2013 (**a**) and 2015 (**b**) analyzed via HSQC NMR. Precision of HSQC integration is about 5%. The two linking patterns, phenylcoumaran (B) and resinol (C), were the smallest proportion of the lignin structure, sometimes well below 10%. There are fluctuations for all genotypes and for both harvests, with NagG10, Sin2, and Rob4 showing the smallest deviations.

**Figure 13:**
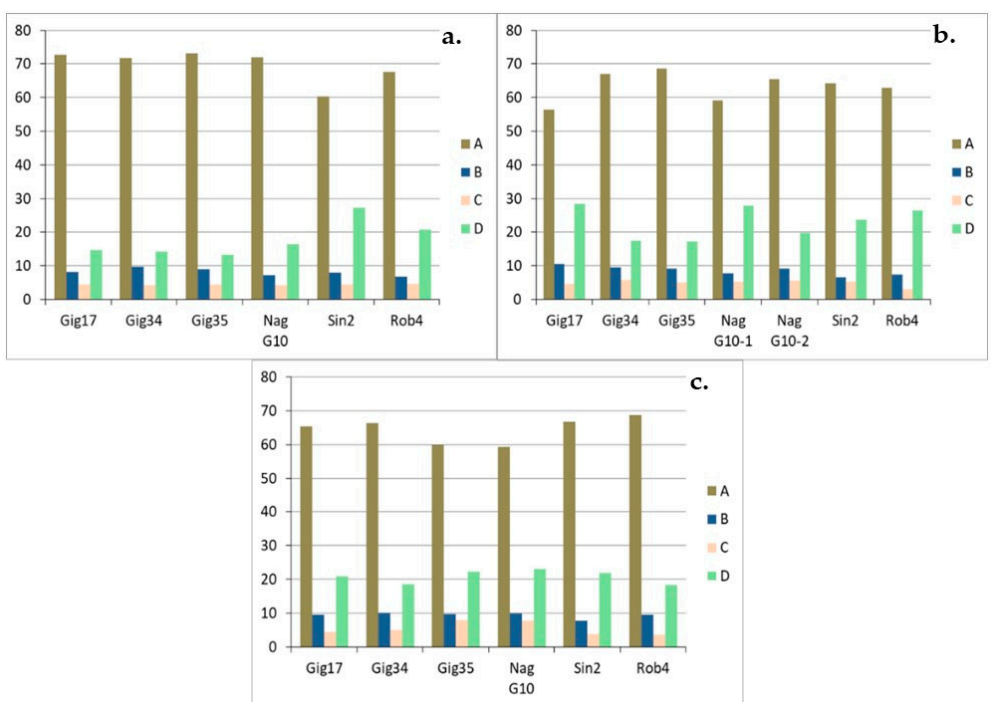
Monolignol ratio (H, G, S in %) of leaf-derived lignins harvested in September (09/15) (**a**), in December (12/14) (**b**) and in April (04/15) (**c**), analyzed via HSQC NMR. NagG10-1 and NagG10-2 are duplicates. Precision of HSQC integration is about 5%.

Two-dimensional (2D) NMR, such as HSQC technique, provides powerful tool for qualitative and quantitative analysis due to much better discrimination of resonances than in 1D NMR. However, 2D NMR peak volumes depend on a number of parameters like relaxation time, pulse sequence delays, pulse angels, off-resonance effects [54]. Therefore, standard 2D HSQC pulse sequence used in this study is only suitable for semi-quantitative analysis of linkages pattern in lignin molecules. Precision of HSQC integration is about 5%. This value should be used for assessing data in Figure 9, Figure 10, Figure 11, Figure 12 and Figure 13. In future studies, we plan to develop HSQC pulse sequence for accurate quantitative measurements.

The monolignol ratio (H/G/S) has been investigated for lignins isolated from the biomass stem-leaf-mixtures (Figure 9a,b), leaf-derived (Figure 10a–c) and stem-derived lignins (Figure 11a–c). 

Precision of HSQC integration is about 5%. For the stem/leaf mixtures harvested in 2013 (Figure 9a) and 2015 (Figure 9b), there is no systematic correlation between genotype and monomer ratio. The ratio varies between the genotypes, but also between the different harvests of the same genotype. For example, *M. nagara* (NagG10), which has more H than S in the 2013 harvest, shows a clearly opposite picture in 2015. In general, G units are the most common ones with around 50%, followed by S units varying between 20 to 30%. The H unit makes up the smallest share with approximately 20%. Duplicates NagG10-12/14-1 and NagG10-12/14-2 show a first indication regarding the reliability of the data. Very recently, statistical studies were performed and published confirming the robustness of this catalyst-free organosolv process to obtain lignins of high purity [31].

For the leaf-derived lignins of all genotypes (Figure 11a–c) an interesting development could be observed: the amount of G units increases significantly from harvest to harvest (ca. 50% in September, ca. 55% in December and ca. 60% in April) for all studied genotypes (except for Gig35).

In contrast, the H portion drops significantly in the same order from approx. 30% in September to approx. 15% in April. There is no remarkable development for the S building block, the share remains almost the same for all genotypes at approx. 20%. For the stem-derived lignins (Figure 11a–c) the share of the G block increases significantly from September to December and remains approximately the same until April. In contrast to the leaf-derived lignins, the composition of the lignin in the stem obviously no longer changes over the winter. For the H and S building blocks, too, there are only deviations between September and December: Both the H and S components decrease somewhat. The duplicates (Gig34-12/14-1 and Gig34-12/14-2) show slight, negligible differences in the compositions, indicating the reproducibility of the applied organosolv procedure. 

In summary, comparing mixtures, leaf- and stem-derived lignins, the leaf-based samples show a high G content of more than 60%, especially in the late harvest in April, which is sometimes well below this value for both stem and mixture samples due to the higher content of stems in mixture samples. In addition, the early harvests in September show a comparatively high proportion of the H building block of around 30% in the leaf samples. Only about 20% were achieved for stem and mixture samples. 

### 3.2. Interunit Linkages Accoroding to NMR-Spectroscopy

In contrast to many previously published NMR spectra, it is striking that there are no signals that indicate sugar residuals, confirming the high purity of the isolated lignins [30,31,44,45,55]. El Hage et al. detected minor amounts of carbohydrates in ethanol organosolv lignin from Miscanthus determined by ^13^C-NMR [34]. In contrast, Vanderghem et al. found up to 14% residual carbohydrates in lignin isolated from Miscanthus via ammonia pretreatment [38]. 

Considering the linkages A, B, C, D (Figure 12a,b), there is no distinct pattern for the lignins isolated from stem/leaf mixtures. Comparing three *M. x giganteus* samples (Gig17, Gig34 and Gig35), harvested in 2013 and 2015, respectively, β-aryl ethers (A) are the most common linkages with approximately 70%, followed by β-unsaturated ester linkages (D) with almost 20%.

The linkage patterns in the leaf-derived lignins (Figure 13a–c) vary considerably, e.g., the β-aryl ethers (A) percentage differs by up to 10%. For all lignins, very high proportion of aryl ether linkages were found. No systematic changes can be determined for the genotypes *M. x giganteus*, *M. nagara* and *M. robustus* (Gig17, NagG10, Rob4), respectively). For the two M. x giganteus samples (Gig34, Gig35), A linkages decrease from September to April harvest whereas D bonds increase. Phenylcoumaran linkages (B) remain relatively constant and resinol bonds (C) increase at least for Gig35. The only exception is the *M. sinensis* (Sin2) where β-aryl ethers (A) rises from September to April whereas resinol (C) and β-unsaturated ester linkages (D) decrease.

**Figure 14:**
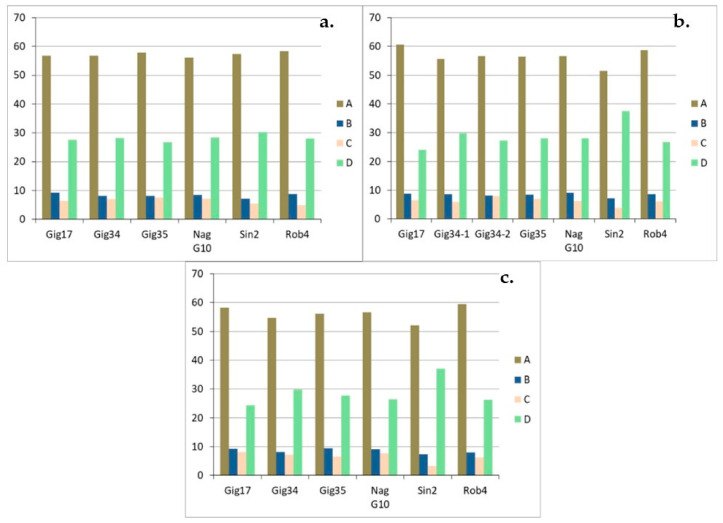
Monolignol linkages (A, B, C, D in %) of stem-derived lignins harvested in September (09/15) (**a**), in December (12/14) (**b**) and in April (04/15) (**c**), due to HSQC NMR. Precision of HSQC integration is about 5%.

The stem-derived lignins show a similarly heterogeneous picture (Figure 14 a–c): here the analysis is also different for all genotypes. *M. x giganteus* (Gig34, Gig35) and *M. nagara* (NagG10) show almost no changes between the different harvests. For *M. sinensis* (Sin2), linkages A decrease from September to April, but D increase significantly. For *M. robustus* (Rob4), B bonds decrease whereas C increase. In contrast, there is no systematic development for the genotype Gig17. 

It can be summarized that the A content is particularly high in leaf-derived lignins at just under 70% and significantly lower in stem and mixture lignins at around 60% and almost 65% respectively. The second most common linkage pattern is D in all isolated lignins, the proportion of which is also strongly dependent on the part of the plant from which the lignin originates. The stem and mixture lignins, for example, have a relatively high share of approximately 20% or more (peak value for *M. sinensis* Sin2 with over 30%). In the leaf-derived lignins, the proportions are significantly lower on average. 

Results presented here for stem versus leaf-based lignins are in good agreement with previously published studies including further data such as crop yields, chemical composition of the biomasses, ash contents [30,31,44,45] and calorific values [44]. If the highest possible lignin content is desired, stem samples should be chosen, specifically from the *M. x giganteus* genotype, which revealed lignin contents up to 27%.

From a European agricultural perspective, the genotype *M. nagara* (NagG10) might be the most suitable for further uses due to its high crop yield and winter hardiness. Studies on *M. nagara* focused the winter cold-tolerance thresholds, cultivation conditions and corresponding yields [56]. Compared to other genotypes, *M. nagara* exhibits a high stability due to very strong stems. Moreover, studies confirmed late mature, fast rhizome formation, a good frost tolerance and a lower leaf loss during winter [57,58,59,60,61,62]. The results regarding the monolignol ratio are in good accordance with HSQC NMR data reported for lignins isolated from Miscanthus crops [31,54,63,64,65,66,67] with the exception of the polysaccharide-lignin-linkages not found here in this study. This is most likely due to the mild catalyst-free organosolv pulping method used.

## 4. Materials and Methods

In the following, the organosolv process, the determination of the chemical composition according to the NREL procedure as well as the HSQC NMR analyses are described. Further details could be found in the corresponding studies reported by our group [30,31,44,45].

### 4.1. Lignin Isolation using a Catalyst-free Organosolv Process

The Miscanthus samples were ground to a particle size of less than 0.5 mm using a ball mill (Fritsch model Pulverisette 6) and a sieving machine (Retsch model AS 200 basic) (Retsch GmbH, Haan, Germany). The digestion method used was previously published in detail [30]. For this purpose, 50 g of the biomass samples were mixed with 500 mL of 80% ethanol solution in a Parr pressure reactor. The ethanolic Organosolv digestion began with heating to 170 °C and holding this temperature for 90 min. After the apparatus had cooled down, the reactor with the liquid-solid mixture was removed and all residue was collected. The biomass was filtered off on a water jet pump and the filtrate was washed with 5 × 50 mL 80% ethanol solution. The mother and washing solutions were collected and then three volumes of deionized water was added and acidified to a pH of 2 with approx. 10 mL 37% hydrochloric acid. The precipitated lignin was centrifuged in, washed five times with deionized water and then freeze-dried. Lignin yields of approx. 20% were achieved.

### 4.2. Chemical Composition of the Biomasses

The determination of the chemical composition of the Miscanthus biomasses was carried out in the laboratories of the Research Institute Bioactive Polymer Systems e.V. (Biopos e.V., Teltow, Germany). Analyses were performed according to the NREL (National Renewable Energy Laboratory) procedures [68].

### 4.3. HSQC NMR Analyses

Approx. 100 mg of the lignin samples were dissolved in 1 mL of deuterated DMSO and transferred to NMR tubes. These were measured on a Bruker NMR Spectrometer Avance III 600 (Bruker BioSpin GmbH, Rheinstetten, Germany) with 4 scans and 16 previous dummy scans. The data from 4000 points were recorded with a spectral width of 7211 Hz and a total acquisition time of 0.28 s. Precision of HSQC integration is about 5%.

## 5. Conclusions

In this review, we show that both, the specific *Miscanthus* genotype factor as well as harvest time have a comparatively small influence on the lignin that is produced. In contrast, the plant component (leaf versus stem) play a more important role to distinguish the lignin structure and interunit linkages. In a previous study, we used principal component analysis (PCA) of various Miscanthus-derived lignin samples to specify the differences between stem and leaf-derived lignins. The projections of infrared data on the first three principal components (PCs) expressed 82% of variance [45]. 

The organosolv process as a digestion method has proven itself in every case: it delivers pure lignins, free of sugar residues. The reproducibility of the catalyst-free process has recently been confirmed in another study [31]. If the differences in the lignins are not sufficient as a criterion for the preference for a particular genotype, one can judge based on the harvest yield or other criteria specifically relating to the plant itself, e.g., the most robust crops regarding weather and climate conditions. 

From an agricultural perspective, the *M. nagara* (NagG10) genotype is the most suitable crop for further material production due to its high harvest yield. When looking at the other agricultural parameters (leaf-to-stem ratio, dry matter, ash content), all genotypes show comparable results. On the other hand, there are clear differences between leaf and stem samples (including dry matter, ash content, etc.). For example, the leaves could be preferred because they ripen more quickly or, on the other hand, the stalks could be used due to their higher calorific values and lower ash contents. 

More precise statements can be made with the help of the NMR data. The H/G/S ratio and linkage pattern within the lignin is obtained. From a chemical perspective, this information can be used, for example, to split specific bonds in a targeted manner, provided the structural units obtained are of interest for further applications. For all Miscanthus genotypes, the G building block is represented most dominantly and H and S fragments vary, especially between harvests. If one considers the linkage pattern as another investigated variable, one finds that β-aryl ethers are in majorities in all lignin types, which is also the case for wood-derived lignins. This is followed by the unsaturated ester and, in significantly lower proportions, phenylcoumaran and resinol. Due to the higher quantity of stems in mixture samples, the variance in the links is significantly greater in mixed and stem lignins than in leaf lignins, where β-aryl ethers also make up a higher proportion. 

The knowledge gained from this work helps with the choice of certain lignin qualities for other chemical applications. Further processing of the already relatively low molecular weight lignin fragments into more defined structures also makes lignin extraction from miscanthus significantly more attractive, for example for applications as bioactive additive in active packaging and medicine. For example, the whole crop application includes direct power generation, fuel production, as well as components for fiber-based hybrid materials such as lightweight concrete [10,69,70,71]. Moreover, cascading use of *Miscanthus* is reported using the biomass successively in integrated processes. As a result, this biomass can be used more effectively, which leads to an increase in added value [72]. 

Within the last decade, wood and grass-derived lignins gained increasing interest [73,74,75,76]. A comprehensive market studies confirmed the urgent demand on sustainable aromatic compounds such as lignin (and its derivatives) to substitute fossil-based aromatic substances in a wide range of applications [77,78]. Due to the aromatic character with inherent number of aliphatic and aromatic hydroxyl groups, lignins are studied as substitutes for diols and polyols for polyurethane synthesis [79,80,81,82] and preparation of phenol-based resins [83]. 

In addition to applications as components in polymer synthesis, lignins are investigated and tested as bioactive additives, in particular as antioxidative [84] and antimicrobial [85] substances. Drug encapsulation gels and hydrogels have been developed using lignins and lignosulfonates [86,87] for applications in tissue engineering and regenerative medicine [88,89,90].

## 6. Future Perspectives

Although the structural analysis of lignins has been a focus of worldwide research activity for years, the rather complex biosynthesis results in numerous interunit linkages that vary from plant to plant resulting in a wide variety of published structures. Even today, hitherto unknown linkages are published, such as tricin structures reported by Lan and colleagues [91]. Thus, it is still a challenge to provide a “specification” for lignin as an industrial raw material. However, studies showed that the *Miscanthus* genotype does not greatly influence the linkage. Thus, fast and reliable analytical methods are important for lignin quality control and assurance. The NMR spectroscopy in principle allows fast characterization. However, as for other spectroscopic methods such as infrared spectroscopy, signal overlap restricts quantitative data interpretation. 

Due to the influx of studies reported within the last decade, chemometric data processing will be critical to solving this problem [31,45,91,92,93,94,95,96,97,98,99]. In particular, methods such as partial component analysis (PCA), linear discriminant analysis (LDA), factorial discriminant analysis (FDA) and partial least squares-discriminant analysis (PLS) were shown to provide data for biomass origin specification of complex structures such as lignin. A problem to be solved in future will be the quantification using 2D NMR. So far, methods such as HSQC are generally limited since pulse sequences are usually optimized for resolution and signal strength, but signal relaxation might not be complete particularly for some slowly relaxing end groups. Here, further efforts are required regarding the underlying mathematical approach.

## Figures and Tables

**Table 1:** Chemical composition according to NREL protocols for leaf-derived Miscanthus. (AIL: acid-insoluble lignin, ASL: acid-soluble lignin, AIR: acid-insoluble residue).

Genotyp	Gig17	Gig34	Gig35	NagG10	Sin2	Rob4
AIL (%)	20.6 ± 0.5	21.1 ± 0.3	19.4 ± 0.9	17.6 ± 0.4	18.6 ± 0.0	18.8 ± 0.1
ASL (%)	5.1 ± 0.3	4.1 ± 0.0	5.1 ± 0.0	4.9 ± 0.0	6.0 ± 0.2	5.7 ± 0.0
AIR (%)	21.8 ± 0.5	22.1 ± 0.4	20.2 ± 1.2	19.2 ± 0.2	19.5 ± 0.0	19.8 ± 0.1
Total lignin (%)	25.5 ± 0.5	25.3 ± 0.5	24.6 ± 0.8	22.5 ± 0.4	24.6 ± 0.2	24.5 ± 0.1
Ash (%)	1.2 ± 0.1	1.1 ± 0.2	0.8 ± 0.2	1.6 ± 0.2	0.9 ± 0.0	1.0 ± 0.1
Glucan (%)	44.8 ± 1.5	45.0 ± 2.3	48.5 ± 1.1	46.3 ± 2.5	43.6 ± 0.3	41.54 ± 0.2
Xylan (%)	28.4 ± 1.8	29.5 ± 0.6	29.4 ± 0.6	29.6 ± 1.1	28.2 ± 0.4	26.5 ± 0.5
Galactan (%)	0.0 ± 0.0	0.0 ± 0.0	0.0 ± 0.0	0.0 ± 0.0	0.4 ± 0.6	1.1 ± 0.0
Arabinan (%)	3.1 ± 0.0	4.1 ± 1.6	3.6 ± 0.3	4.7 ± 0.1	4.4 ± 0.0	3.6 ± 0,0
Mannan (%)	0.0 ± 0.0	0.0 ± 0.0	0.0 ± 0.0	0.0 ± 0.0	2.3 ± 0.0	2.3 ± 0,0
Dry matter (%)	92.5	91.2	91.2	91.4	91.9	92.6
Total ash (%)	4.5	6.8	5.2	6.4	5.4	5.8

**Table 2:** Chemical composition according to NREL protocols for stem-derived Miscanthus. (AIL: acid-insoluble lignin, ASL: acid-soluble lignin, AIR: acid-insoluble residue).

Genotype	Gig17	Gig34	Gig35	NagG10	Sin2	Rob4
AIL (%)	21.2 ± 0.0	21.0 ± 0.4	22.0 ± 0.1	21.3 ± 0.1	19.2 ± 0.1	19.2 ± 0.1
ASL (%)	4.7 ± 0.3	4.3 ± 0.0	5.0 ± 0.1	4.5 ± 0.1	5.7 ± 0.0	5.3 ± 0.2
AIR (%)	22.4 ± 0.2	22.3 ± 0.6	22.5 ± 0.1	21.8 ± 0.1	19.8 ± 0.2	19.8 ± 0.1
Total lignin (%)	26.0 ± 0.4	25.2 ± 0.4	27.0 ± 0.2	25.8 ± 0.1	24.9 ± 0.1	24.5 ± 0.1
Ash (%)	1.3 ± 0.1	1.0 ± 0.0	0.6 ± 0.0	0.5 ± 0.1	0.7 ± 0.1	0.6 ± 0.1
Glucan (%)	50 ± 0.6	50.5 ± 0.9	49.6 ± 0.4	47.1 ± 1.4	48.2 ± 3.0	45.7 ± 0.3
Xylan (%)	27.4 ± 2.8	26.2 ± 0.5	23.6 ± 0.0	23.9 ± 0.5	28.7 ± 1.7	25.6 ± 0.3
Galactan (%)	0.0 ± 0.0	0.0 ± 0.0	0.2 ± 0.4	0.0 ± 0.0	0.0 ± 0.0	0.0 ± 0.0
Arabinan (%)	1.9 ± 0.5	2.0 ± 0.3	1.8 ± 0.4	1.8 ± 1.6	2.9 ± 0.2	2.1 ± 0.2
Mannan (%)	0.0 ± 0.0	0.0 ± 0.0	1.1 ± 1.6	0.0 ± 0.0	2.3 ± 0.1	2.3 ± 0.0
Dry matter (%)	92.2	92.2	92.1	93.0	92.8	92.7
Total ash (%)	2.5	3.1	2.4	1.8	3.4	2.2
